# Managed honeybees and South American bumblebees exhibit complementary foraging patterns in highbush blueberry

**DOI:** 10.1038/s41598-021-87729-3

**Published:** 2021-04-14

**Authors:** M. Cecilia Estravis-Barcala, Florencia Palottini, Ivana Macri, Denise Nery, Walter M. Farina

**Affiliations:** 1 Laboratorio de Insectos Sociales, Departamento de Biodiversidad y Biología Experimental, Facultad de Ciencias Exactas y Naturales, Universidad de Buenos Aires, Buenos Aires, Argentina; 2 Instituto de Fisiología, Biología Molecular y Neurociencias (IFIBYNE), CONICET-Universidad de Buenos Aires, Buenos Aires, Argentina; 3 Instituto de Ingeniería Rural, Centro de Investigación de Agroindustria (CIA), Instituto Nacional de Tecnología Agropecuaria (INTA), Castelar, Buenos Aires Argentina

**Keywords:** Zoology, Ecology

## Abstract

Despite *Apis mellifera* being the most widely managed pollinator to enhance crop production, they are not the most suitable species for highbush blueberries, which possess restrictive floral morphology and require buzz-pollination. Thus, the South American bumblebee *Bombus pauloensis* is increasingly managed as an alternative species in this crop alongside honeybees. Herein, we evaluated the foraging patterns of the two species, concerning the potential pollen transfer between two blueberry co-blooming cultivars grown under open high tunnels during two seasons considering different colony densities. Both managed pollinators showed different foraging patterns, influenced by the cultivar identity which varied in their floral morphology and nectar production. Our results demonstrate that both species are efficient foragers on highbush blueberry and further suggest that they contribute positively to its pollination in complementary ways: while bumblebees were more effective at the individual level (visited more flowers and carried more pollen), the greater densities of honeybee foragers overcame the difficulties imposed by the flower morphology, irrespective of the stocking rate. This study supports the addition of managed native bumblebees alongside honeybees to enhance pollination services and emphasizes the importance of examining behavioural aspects to optimize management practices in pollinator-dependent crops.

## Introduction

Despite honeybees being the most adaptable and widely managed pollinator to enhance crop production^1^, the global stock of *Apis mellifera* colonies is growing slower than agricultural demands for pollination services^2^. With the expansion of areas cultivated with pollinator-dependent crops around the globe^3^, the contribution of wild insects to pollination has been recognized for a wide variety of annual and perennial species^4,5^. However, the abundance and diversity of pollinator assemblages vary in different agroecosystems, and growers often rely on managed honeybees for pollination services. Therefore, there has been a growing interest in managing alternative species, such as bees of the genera *Osmia*, *Bombus,* and *Megachile*^6,7^. Bumblebees have been proven to be efficient pollinators of greenhouse as well as of outdoor crops^8,9^. Furthermore, an integrated approach combining native bees with honeybees can enhance pollination services, due to complementarity in their foraging behaviour^10,11^.

Highbush blueberry *Vaccinium corymbosum*, though native to eastern North America, is now cultivated globally. Blueberry global production increased 57% between 2013 and 2018^12^. In South America acreage has expanded steadily in the last decade, with Argentina accounting for 20 thousand tons in 2018^13^. In these latitudes, southern cultivars are being grown, which were originally developed in the 1980s in Florida where highbush blueberries could not meet their high chilling requirement^14^. The numerous cultivars resulting from selective breeding, present varying degrees of pollinator dependency, yet all benefit from cross-pollination and hence two or more cultivars are usually planted in adjacent rows^15,16^. Also, blueberry cultivars exhibit diverse floral morphologies that may affect visitation rates and behaviour of pollinators^17^. Blueberry flowers are small, with urceolate corollas which limit the access of visitors to the basal nectaries. In addition to its accessibility, intraspecific variability of the quantity and quality of nectar could modify the attractiveness of different cultivars to bees. Blueberry flowers are also visited for their pollen. Their poricidal dehiscent anthers require buzz-pollination, i.e., the behaviour of vibrating flowers to release great amounts of pollen. While many wild bees present in blueberry native distribution are able to buzz-pollinate the anthers^18^, honeybees do not have this ability and are considered less effective pollinators^19,20^. Therefore, to ensure pollination, growers invest up to 12 honeybee colonies per ha^16,18^.

In contrast, bumblebees possess many characteristics which give them great pollination potential^21^. *Bombus* spp. are able to ‘buzz’ flowers^22^, and their large body size enables them to carry more pollen grains adhered on their body hairs than honeybees, which can then be transferred to flowers’ stigmas during visits^23^. All these attributes are well suited for pollination of blueberry. In fact, wild and commercially reared *Bombus* are considered more effective pollinators of blueberry than *A. mellifera*^19,20,24,25^. Notwithstanding the effectiveness of *B. impatiens* as a managed pollinator, the worldwide trade of bumblebee colonies has raised concern about the potential impact of invasive species on native populations^26,27^ and special attention has turned to native species.

In Argentina and the Andean region of South America, the native bumblebee *Bombus pauloensis* (sin. *Bombus atratus*) is an alternative pollinator increasingly managed in blueberry plantations among other crops. Commercially reared colonies are introduced along with honeybee hives, which nevertheless remain the main managed pollinator. Little has been documented, however, about the impact of supplementing honeybees with other managed species in blueberry fields. Although some studies show a positive effect of *B. pauloensis* colonies in the fruit production^28–30^, there are few studies evaluating the foraging behaviour of this native species in different cultivars which may differ in their attractiveness to pollinators.

Our aim was to evaluate the foraging patterns of *A. mellifera* and *B. pauloensis* at an individual and population level, in two blueberry co-blooming cultivars frequently grown together, ‘San Joaquín’ and ‘Emerald’. As a first step, we characterized their floral morphology and nectar reward. Secondly, we assessed the density of bees on both cultivars (grown under open high tunnels), considering different colony stocking rates. Thirdly, we evaluated the foraging patterns of the managed pollinators, in terms of number of flowers visited, type of resource exploited and floral constancy in each cultivar. We also quantified the amount of blueberry pollen carried on their bodies to estimate the potential pollen transfer between flowers during foraging bouts.

## Results

### Characterization of blueberry cultivars

The two blueberry cultivars studied varied in their floral morphology (Supplementary Table S1). The MANOVA indicated overall differences between cultivars (Wilk’s Lambda, F_3,105_ = 20.997, p < 0.001). Follow-up univariate Anovas indicated that ‘San Joaquin’ flowers exhibited a smaller corolla throat diameter than ‘Emerald” ones (F_1,107_ = 24.8, p < 0.001), with a shorter (F_1,107_ = 7.0, p < 0.01) and narrower corolla (F_1,107_ = 63.0, p < 0.001).

Also, both cultivars differed in their nectar availability (Supplementary Fig. S1a). ‘San Joaquin’ flowers offered higher amounts of nectar than ‘Emerald’ ones (mean ± SE, *SJ*: 4.0 ± 0.3 μl/flower, N = 47; *EM*: 2.9 ± 0.2 μl/flower, N = 59; χ^2^_1_ = 8.32, p < 0.01), although both were similar in sugar concentrations (mean ± SE, *SJ*: 18.6 ± 1.0% w/w, N = 33; *EM*: 20.3 ± 0.8% w/w, N = 32; Supplementary Table S2). Cultivars also differed in nectar production (Supplementary Fig. S1b). ‘San Joaquin’ unvisited flowers offered higher volumes than ‘Emerald’ (*EM* vs *SJ*: LR = 18.11, p < 0.001). Besides, bagged flowers of both cultivars showed an increase in their nectar volume during the 3 days evaluated (flower age: LR = 98.95 p < 0.001; post hoc comparisons: 1-day vs 2-day old: p < 0.001; 2-day vs 3-day old p < 0.001; 1-day vs 3-day old*:* p < 0.001). Furthermore, we found no significant differences between the estimated sugar concentration of unvisited flowers in 2019, (mean ± SE, *SJ*: 59 ± 1% w/w, N = 47; *EM*: 60 ± 1% w/w, N = 60; Supplementary Table S2).

### Foraging patterns at population level

**Figure 1:**
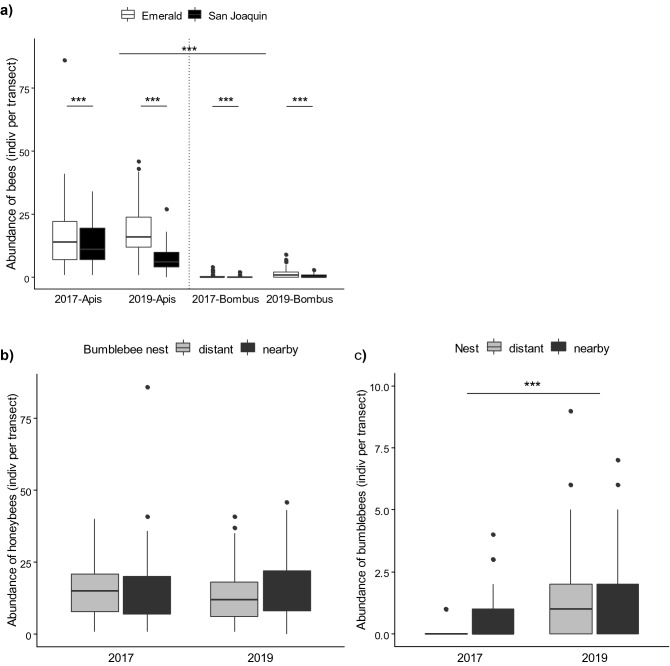
Foraging patterns of *Apis mellifera* and *Bombus pauloensis* at population level on ‘Emerald’ and ‘San Joaquin’ cultivars. **(a**) Abundance of individuals per transect. Honeybees far outnumbered bumblebees but did not vary between years. (**b**,**c**) Abundance of honeybees (**b**) and bumblebees (**c**) per transect, considering the proximity to a bumblebee nest. The density of honeybees differed between cultivars but was not affected by the proximity to a *Bombus* colony. The number of bumblebee foragers increased with a high stocking density of colonies but was not affected by the presence of a nest nearby. Boxplots show the median and interquartile range (IQR), with whiskers showing the maximum value within 1.5 IQR, and individual points mark values outside this range. Asterisks indicate statistical differences (***, p < 0.001). (N = 369 transects).

When we assessed the number of managed pollinators per transect, honeybees far outnumbered bumblebees foraging on blueberry flowers (Fig. 1a). There was a significant interaction between species and year (LRT 53.8, p < 0.001), while honeybee densities did not differ between both seasons (2017: 15.4 ± 1.0 indiv transect^−1^; 2019: 14.6 ± 0.7 indiv transect^−1^; post hoc comparison *2017 vs 2019*: F.ratio_1,730_ = 43.7, p = 0.3673), the density of bumblebees was indeed modified by the year (post hoc comparison *2017 vs 2019*: F.ratio_1,730_ = 43.7, p < 0.001). Bumblebees abundance in 2019 showed a > threefold increase, consistent with a higher stocking of nests (2017: 0.4 ± 0.1 indiv transect^−1^; 2019: 1.2 ± 0.1 indiv transect^−1^). As for the densities on each cultivar, there was a higher number of bees foraging on ‘Emerald’ flowers (*EM* vs *SJ*: LR = 32.72, p < 0.001). When we considered the distribution of bumblebee colonies, the number of pollinators was not affected by the proximity of a bumblebee nest (Fig. 1b,c; Supplementary Table S2).

### Foraging patterns at individual level

**Figure 2:**
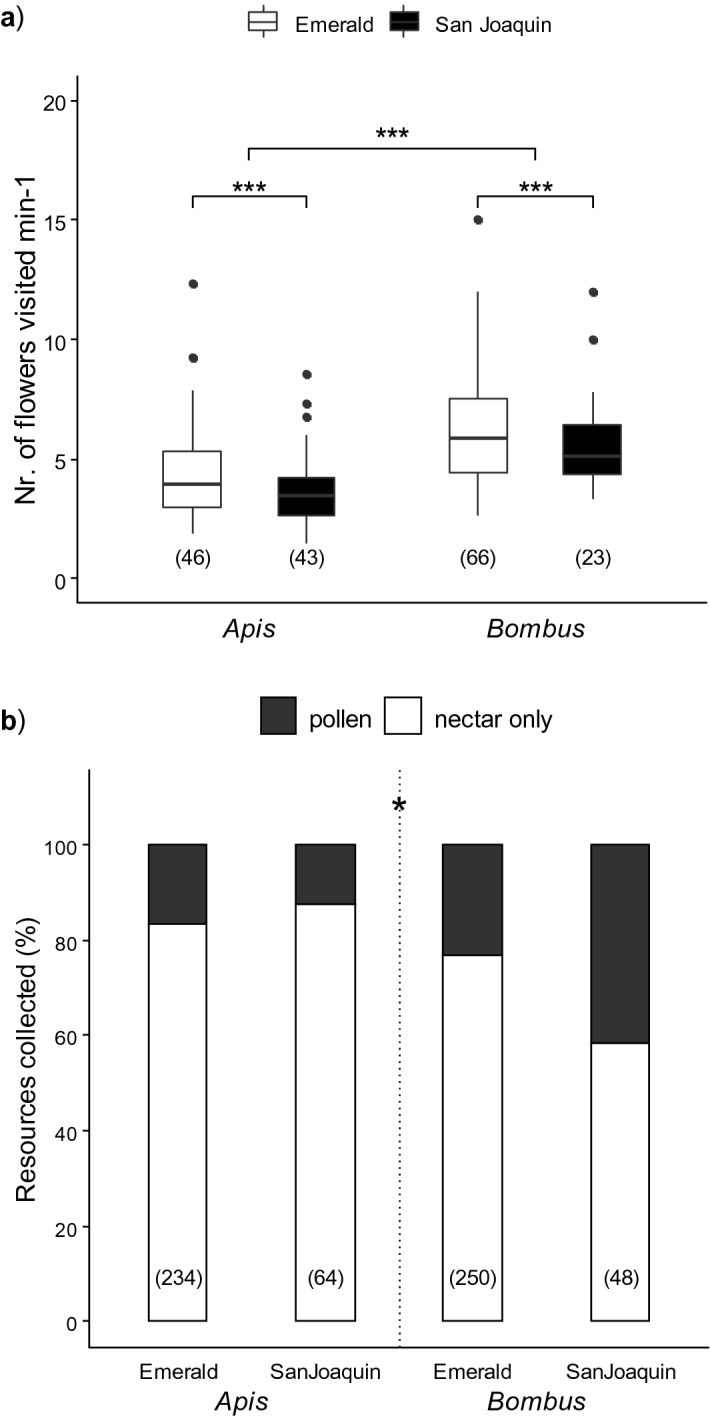
Foraging patterns of *Apis mellifera* and *Bombus pauloensis* on ‘Emerald’ and ‘San Joaquin’ cultivars. **(a**) Number of flowers visited per minute by both species. Bumblebees visited more flowers than honeybees and ‘Emerald’ flowers were more frequently visited by both species. Boxplot shows the median and interquartile range (IQR), with whiskers showing the maximum value within 1.5 IQR, and individual points mark values outside this range. (**b**) Resources foraged by both species (EM: Emerald; SJ: San Joaquin). Bumblebees foraged for pollen in a greater proportion on both cultivars. Asterisks indicate statistical differences (***, p < 0.001). Sample size indicated in brackets.

When we monitored honeybees and bumblebees foraging on both cultivars, we found significant differences between both species (Fig. 2). *B. pauloensis* visited a higher number of blueberry flowers per minute than *A. mellifera* (Fig. 2a, mean ± SE, *Bombus*: 6.1 ± 0.3 flowers min^−1^; *Apis*: 4.1 ± 0.2 flowers min^−1^; *Apis* vs *Bombus*: LR = 39.37, p < 0.001). In addition, ‘Emerald’ flowers were more frequently visited by both species than ‘San Joaquin’ inflorescences (*EM* vs *SJ*: LR = 7.47, p < 0.01).

We also found differences in the resources collected (Fig. 2b). Even though the majority of individuals foraged exclusively for nectar on both cultivars, there was a significant interaction between pollinator species and cultivar (LRT = 5.40 p = 0.0202). While the proportion of nectar-foraging honeybees did not differ between cultivars (post hoc comparison *EM vs SJ*: F.ratio_1,589_ = 0.42, p = 0.5193), a higher proportion of bumblebees foraging on ‘San Joaquin’ carried corbicular pollen (post hoc comparison *EM vs SJ*: F.ratio_1,589_ = 6.60, p = 0.0105). On the other hand, the resource foraged was not affected neither by the year nor by the time of day (Supplementary Table S2). All visits were legitimate as we observed no floral damage (indicating nectar robbery).

**Figure 3:**
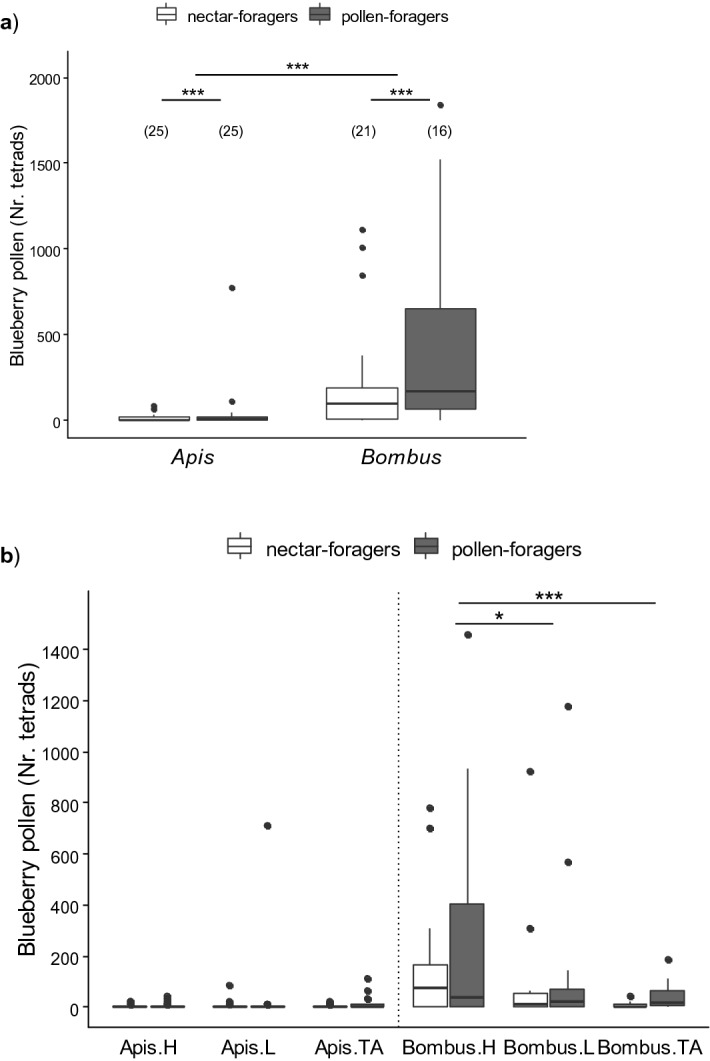
Blueberry pollen on *Apis mellifera* and *Bombus pauloensis.* **(a)** Tetrad counts on the body of foragers. Bumblebees carried ten times more blueberry pollen on their body than honeybees, and significantly more tetrads were found on pollen- than on nectar-foragers. (**b**) Tetrad counts on head (H), legs (L) and thorax-abdomen (TA) of foragers. While bumblebees carried more blueberry pollen on their head, the amount of tetrads on honeybees did not differ between the regions evaluated. Boxplot shows the median and interquartile range (IQR), with whiskers showing the maximum value within 1.5 IQR, and individual points mark values outside this range. Asterisks indicate statistical differences (*, p < 0.05; ***, p < 0.001). Sample size indicated between brackets.

Regarding the pollen collected from the bodies of pollinators, *B. pauloensis* foragers carried approximately 10 times more blueberry pollen than honeybees (Fig. 3, Supplementary Table S2). Pollen-foragers of both species presented more blueberry pollen on their bodies (across all three evaluated areas) than nectar-foragers (Fig. 3a, *Apis* vs *Bombus*: p < 0.001; *Nectar- vs Pollen-foragers*: p < 0.01). The analysis of pollen loads on the main regions of the body evaluated revealed a significant interaction between species and region (Fig. 3b, species * body region, LR = 12.87, p < 0.001). While the number of tetrads carried by honeybees did not differ among the three body parts evaluated, bumblebees carried significantly more blueberry pollen on their head (post hoc comparison for *Bombus*: Head vs Legs: p < 0.05, Head vs Thorax-abdomen: p < 0.0001). The amount of pollen on the head of bumblebees doubled that on their legs and surpassed over 8 times that on the thorax-abdomen region (mean ± SE, Head: 202 ± 53 tetrads, Legs: 102 ± 42 tetrads, Thorax-Abdomen*:* 23 ± 7 tetrads).

**Figure 4:**
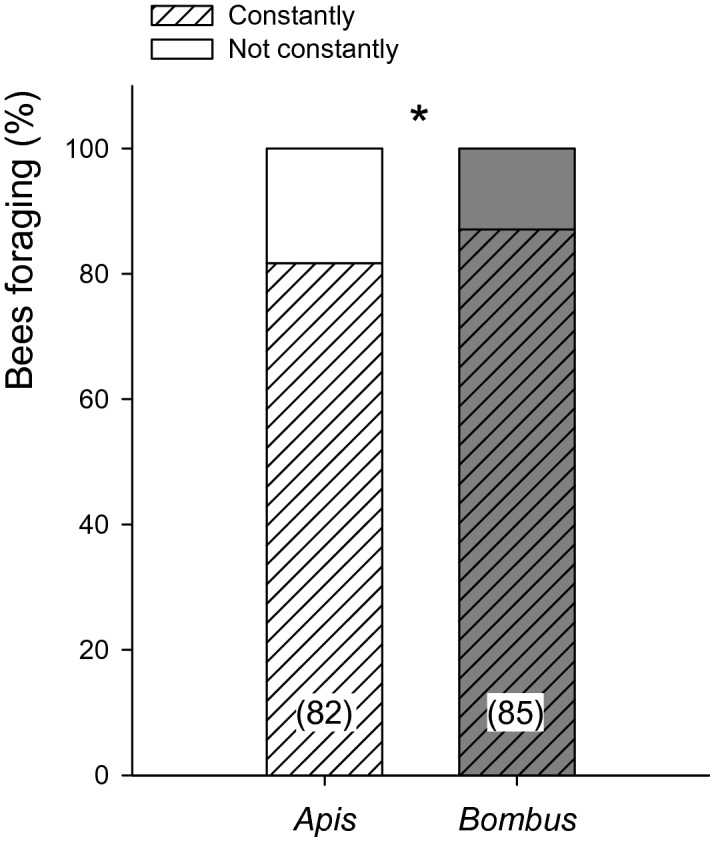
Floral constancy of *Apis mellifera* and *Bombus pauloensis*. The percentage of individuals foraging exclusively on the same cultivar is represented with stripes (constantly). Full bars represent the percentage of bees shifting between cultivars (not constantly). Both species exhibited high floral constancy, regardless of the cultivar, but honeybees were more likely to switch between cultivars than bumblebees. Asterisks indicate statistical differences (*, p < 0.05). Sample size indicated in brackets.

To assess floral constancy, we monitored a total of 82 honeybees and 85 bumblebees sequentially visiting on average (mean ± SE) 10.7 ± 0.9 (3–41) and 18.2 ± 1.7 (3–73) blueberry flowers, respectively. When we evaluated the movement of bees and the potential transfer of pollen between cultivars, we found that both species exhibited high fidelity to the same cultivar (≥ 80%), regardless of its identity (Fig. 4, Supplementary Table S2). However, the proportion of bumblebees constantly foraging on a given cultivar was significantly higher than the number of honeybees (Apis vs Bombus: LR = 4.29 p = 0.0384). That is, honeybees were more likely to switch between ‘Emerald’ and ‘San Joaquin’ flowers than bumblebees.

## Discussion

Our study shows that introduced honeybees and managed native bumblebees differed in their foraging patterns on highbush blueberry cultivars ‘Emerald’ and ‘San Joaquin’ under open high tunnels, both at the population and at the individual level. The abundance of *Apis mellifera* and *Bombus pauloensis* visiting blueberry flowers and their individual behaviour was influenced by the identity of the cultivar, which varied in their floral morphology as well as in the nectar production. At the population level, honeybees far outnumbered bumblebees foraging on blueberry flowers, and their density was not affected neither by the stocking rate nor by the proximity of a bumblebee nest. In contrast, a higher stocking of bumblebee colonies in the second season resulted in an increase of foragers on the crop. At the individual level, *B. pauloensis* visited a higher number of flowers per minute than *A. mellifera*, and a higher proportion of them collected pollen throughout the day. Also, bumblebees carried 10 times more blueberry pollen adhered on their bodies than honeybees, particularly pollen- foragers. Finally, though both pollinator species exhibited high flower constancy, honeybees were more likely to switch between cultivars than bumblebees, thus promoting cross-pollination.

In the open high tunnels studied, a semi-protected system including a plastic cover but no side walls, both pollinator species were found foraging on ‘Emerald’ and ‘San Joaquin’ plants and showed no orientation problems to return to their colonies. Honeybees were the dominant visitors of the crop in both years, exceeding by far the density of *B. pauloensis* on blueberry flowers (more than tenfold increase in the two seasons evaluated). Surprisingly, the number of honeybee foragers was not modified by the year, even though in 2019 the stocking rate of hives was 3.5 times higher than in 2017. Although in 2019 growers stocked the field with 20 hives/ha, this did not translate into more honeybees foraging on the crop. Our results are consistent with other studies reporting a poor correlation between colonies stocking rate and abundance in blueberry fields^18,25^, implying that honeybees could be foraging with less intensity or on alternative flora in the surroundings, although in July there are no abundant flowering species in the region. In contrast, increasing the stock of *B. pauloensis* colonies by a factor of 1.5 in 2019 resulted in a significant increment of bumblebee foragers on blueberry. Our results support the stocking rate of 5–6 colonies /ha recommended for managed *B. pauloensis* in highbush blueberry^28^ as well as for managed *Bombus impatiens* colonies in lowbush blueberry^24^. Yet, since most growers plant different blueberry cultivars to optimize production over the season, the stocks of pollinators could be underestimated in our study, since surrounding cultivars were not in bloom at the time. Secondly, the abundance of both managed species was not affected by the proximity of a bumblebee nest and no antagonistic interactions between them were witnessed during the 2-year study. On the other hand, the abundance of honeybees and bumblebees was strongly related to identity of the blueberry cultivar. In the two seasons, both species were more frequently found foraging on ‘Emerald’. Such preference could be explained by the higher number of open flowers offered by ‘Emerald’ plants in both years, and the fact that their floral rewards would be more accessible to visitors (due to larger corolla openings), even though ‘San Joaquin’ flowers offered greater volumes of nectar. In this regard, our results are consistent with the findings of Courcelles and collaborators^17^ in northern highbush cultivars, supporting the hypothesis that the corolla opening is the main morphological determinant of visit rates of bees. Though both cultivars produced ample amounts of nectar over their flower lifespan, ‘San Joaquin’ flowers presented higher nectar volumes and possessed narrower corolla openings which may prevent evaporation. On the other hand, we found no significant differences in the sugar content of both cultivars neither when we assessed the nectar standing crop nor in unvisited flowers.

Both managed pollinators differed in their individual foraging behaviour. *B. pauloensis* visited a higher number of blueberry flowers per unit time than *A. mellifera*. Such behaviour is consistent with the fact that their longer proboscises enable them to collect nectar faster from deep corollas. Faster flower handling times have been reported for other *Bombus* species when contrasted with honeybees foraging on lowbush blueberries^19,24^. Given blueberry flowers are nectariferous, more than 85% of honeybees foraged exclusively for nectar on both cultivars during the two seasons. The low proportion of pollen-foragers could be due to, on the one hand, its low crude protein content, which is below honeybees’ nutritional requirement (13.9%)^31^, and on the other hand, the difficulty of honeybees to harvest this resource. Because of their inability to buzz-pollinate, honeybees forage mostly for nectar on blueberry flowers^24,32^. Nectar-foragers on *Vaccinium* species were more abundant than pollen-foragers and contributed to the successful transfer of pollen although they were less effective pollinators than the latter^33^. In such scenario, legitimate non-buzzing honeybees can perform efficiently, passively collecting pollen while foraging for nectar. On the contrary, though most *B. pauloensis* foraged for nectar, a higher proportion of them actively foraged for pollen, especially when visiting ‘San Joaquin’ plants. Thus, *B. pauloensis* could contribute to pollination based on the fast flower handing time and their ability to effectively harvest blueberry pollen, as other *Bombus* species were efficient pollinators on a per-visit basis^20^.

Bees foraging behaviour determines the amount of pollen grains which adhere on the hairs of their bodies and therefore can be later deposited on the stigma of another flower. Our results revealed that *B. pauloensis* foragers, which possess a larger body surface and denser body hairs than honeybees, carried 10 times more blueberry pollen tetrads on their bodies than *A. mellifera*. The amounts of blueberry tetrads resulted lower than the ones reported by other authors^32,34^, which might be due to a lower sample size and minimum variation in the protocol applied. Despite these differences, our study allowed us to compare the relative amounts of pollen carried by pollen and nectar foragers. Pollen-foragers of both species retained significantly more pollen on their bodies than nectar-foragers denoting that the resource foraged influences the way in which bees manipulate the flower and ultimately impacts the potential pollen transfer between flowers. However, greater amounts of pollen on the bodies of bees actively collecting this resource could be offset by its lower adherence to the stigmas or germinative aptitude as result of the packing behaviour^35^. Since the morphology of blueberry flowers limits contact of foragers with the stigma, we analysed the pollen counts of three main body parts (head, legs and thorax-abdomen). While the number of tetrads carried by honeybees did not differ among the body parts evaluated, bumblebees carried significantly more blueberry pollen on their head. Nectar-gathering bees insert their head almost completely inside the corolla, suggesting the potential for pollen transfer as they contact the stigma in order to reach the nectaries. The ability of bumblebees to sonicate anthers to release considerable amounts of pollen^22^, and their positioning on the flower with their large head almost completely covering the corolla opening, could explain the higher amounts of pollen on this body part, suggesting the existence of ‘safe spots’ carrying pollen within the head after grooming behaviour.

Bees carrying pollen from a different cultivar on their bodies can cross-pollinate blueberry flowers and enhance fruit production. Our results show that honeybees and bumblebees exhibited high fidelity to the same cultivar. However, honeybees were more likely to switch between cultivars than bumblebees, thus promoting cross-pollination, though the low amount of pollen quantified on the body of honeybees, particularly nectar-foragers, would require a high number of visits for an effective pollination. Flower constancy is common in social bees^36^ and has been usually evaluated in flower arrays of different plant species. However, in dimorphic hybrid crops honeybees have shown fewer transition flights between parental lines with increasing dimorphism, hindering the transfer of pollen between cultivars^37,38^. *Bombus* foragers were also more likely to switch between species only if flowers had strong similarity in appearance^39^. Although ‘Emerald’ and ‘San Joaquin’ varied in their floral morphology and nectar production, the constancy of honeybees and bumblebees was not modified by the cultivar. The fidelity of bees could indicate a trade-off between the amount of nectar, its accessibility, and the number of blossoms. Moreover, both cultivars might vary in the floral scent or the nutritional quality of the floral rewards, aspects which could affect their attractiveness to bees^40^, but which were not evaluated in the present study. Intraspecific variation of floral volatiles and secondary metabolites from nectar and pollen has been demonstrated for some blueberry cultivars^41,42^ and could influence pollinators foraging efficiency.

While positive effects of adding a second managed pollinator have been reported for fruit and nut orchard production^11,43^, studies assessing the impact of supplementing honeybees with other managed species in highbush blueberry are scarce. Improvement of fruit yield and quality has been reported for blueberry plots stocked with *A. mellifera* and *B. pauloensis* colonies^28–30^. However, this is the first report to document the foraging patterns of both managed pollinators over two years with different stocking densities in a mixed plantation, considering the variability of the floral morphology and nectar reward of two co-blooming cultivars. Managing complementary pollinators becomes relevant across blueberry production regions outside its native distribution, where wild bee communities can be limited to only a few species. The contribution of wild bees to blueberry fruit set has been demonstrated within its native range, where wild bees accounted for up to 30% of visits^4,16,44^. Although in those latitudes blueberry blooms in spring and is visited by many native bees species, in other production regions (including our study system) early winter-blooming cultivars are grown to produce berries that can be harvested for the export market before other high-chill cultivars grown in the northern hemisphere^14^. It is worth mentioning that 39% blueberry worldwide production in 2018 relied on regions outside its native range, showing a positive trend during the last five years^12^. In such agroecosystems, many of which are intensively managed, wild bee communities are probably scarce, but could be promoted by adding flower strips or hedgerows, which provide nesting and foraging resources for wild pollinators before and after crop flowering^45^. Alternatively, the introduction of managed native bumblebees can supplement the pollination services provided by honey bees where wild bees are less abundant.

Overall, our results demonstrate that *A. mellifera* and the native bumblebee *B. pauloensis* are both efficient foragers on highbush blueberry under open high tunnels and further suggest that they contribute positively to its pollination in complementary ways. While bumblebees exhibited a higher visitation frequency of blueberry flowers and were more effective in collecting their pollen throughout the day, the greater worker population of honeybees was able to overcome the difficulties imposed by the specialized flower morphology of this crop. Therefore, the present study supports the addition of managed native bumblebees alongside honeybees to enhance pollination services and emphasizes the importance of integrating ecological knowledge about floral traits variation among cultivars and their impact on bees foraging behaviour, to optimize management practices in pollinator-dependent crops.

## Materials and methods

### Study site and managed pollinators

Field studies were performed during the highbush blueberry blooming seasons in July 2017 and July 2019, in a plantation near Gobernador Virasoro (27°56′44.98″S, 56°5′30.29″W), province of Corrientes, in the northeast region of Argentina. Southern highbush blueberry cultivars ‘Emerald’ (EM, *V. corymbosum* × *V. darrowi*) and ‘San Joaquin’ (SJ, *V. corymbosum* hybrid) were grown under open interconnected high tunnel structures of 10 m wide, 50 m long and 3.5 m tall, with metal frames and a plastic cover but no side walls to facilitate ventilation. Under each tunnel cultivars were planted in two sets of 3 rows (1EM: 1SJ: 1EM, 1.7 m apart from each other), separated by an intermediate street (2.7 m wide). We selected a total of 306 rows encompassing a total of 25,212 plants of the same age, which occupied 2.65 ha within two field zones (of 1 ha and 1.65 ha, respectively). Both zones, which were ~ 400 m apart within the same plantation, separated by tree windbreaks and subjected to the same management practices in terms of irrigation, fertilizer and pesticide applications.

Commercial *Apis mellifera* Langstroth hives (20,000 worker strength) and *Bombus pauloensis* colonies (100–120 worker strength) were introduced in the field with different stocking densities in the two seasons. In 2017, a total of 33 honeybee hives were located 10 m apart from the blueberry plants, at a stocking density of 5.6 hives /ha. On the other hand, 24 bumblebee nests were placed within the rows (1 colony /24 rows), so that the bumblebee stocking density achieved was of 4 colonies /ha. In 2019, stocking densities were considerably higher. In that year, 115 honeybee hives were placed on one end of blueberry rows (20 hives /ha), while a total of 36 bumblebee nests were located at the opposite end, (6 colonies/ha).

### Characterization of blueberry cultivars

‘Emerald’ and ‘San Joaquin’ are early winter-blooming cultivars, and normally reach full bloom in late-July in northeast Argentina. Firstly, we characterized both cultivars in terms of the floral morphology and nectar reward (see Supplementary methods). Though their phenology overlapped during the two seasons, ‘Emerald’ bushes possessed more open flowers (mean ± SE, 2017: *EM:* 45.7 ± 5.6 open flowers per plant, *SJ*: 18.0 ± 3.0 open flowers per plant, N = 32 plants/cultivar; 2019: *EM:* 59.8 ± 5.8 open flowers per branch, *SJ*: 40.1 ± 3.2 open flowers per branch, N = 32 plants/cultivar).

### Foraging patterns at population level

We sampled the density of honeybees and bumblebees on the crop, by daily recording the number of individuals foraging on flowers of each cultivar during 4 and 5 consecutive days in 2017 and 2019, respectively, from 9:30 to 16:30 h, the bees’ most active hour. We counted the total number of bees along 50-m-transects in both zones in the two years (N = 126 transects in 2017, N = 243 in 2019). Observations were done both during the morning and the afternoon in case there was a bias in the timing of pollinator activity. We also considered the distribution of bumblebee colonies, distinguishing between those transects close to a bumblebee nest (within 5 rows) from those distant from one.

### Foraging patterns at individual level

We studied the foraging behaviour of *A. mellifera* and *B. pauloensis* by monitoring individual bees during their sequential foraging visits from the moment they landed on a blueberry flower. A total of 89 individuals of each species were monitored during four consecutive days in 2017. Firstly, we registered the number of flowers visited by each bee, recording the duration of the visits, the cultivar and the time of day (Morning, 9–13 h, or Afternoon, 13–16 h) until the observer lost sight of the focal bee.

To evaluate any differences in the foraging preferences of both species, we recorded the resource exploited on each cultivar, during morning and afternoon visits of both years (N = 149 individuals per species per year). We categorized bees with pollen in the corbiculae as pollen-foragers and individuals extending their proboscis and without pollen in the corbiculae as nectar-foragers. Additionally, we considered the visit as ‘legitimate’ (if the bee inserted its proboscis through the apical opening of the corolla) or ‘robbing’ (if the bee inserted the proboscis reusing a slit in the corolla, without contacting the stigma).

To assess the pollen carried by bees on their bodies, we collected 50 honeybee and 37 bumblebee foragers from the blueberry field in 2019. Individuals were captured in vials and immediately frozen until processing at the laboratory. Following the methodology described by Hoffman and collaborators^35^, each bee was dissected into three main body parts: head, thorax-abdomen (wings removed) and legs (see Supplementary methods). Since the resource collected influences the floral structures contacted, we distinguished between pollen- and nectar-foragers. If the forager carried pollen loads on the corbiculae, it was categorized as pollen-forager and the hind legs were removed and discarded. Individuals without pollen in the corbiculae were considered nectar-foragers.

Finally, since blueberry production benefits from cross-pollination^46^, we evaluated the floral constancy of foragers. Bees were monitored during their sequential visits for a maximum of 10 min, recording if they switched between cultivars or not, to calculate the percentage of bees that showed constancy on each cultivar. If the observer lost sight of the focal bee before it visited at least three flowers, the observation was excluded from the analysis.

### Statistics

All analyses were performed with R v3.6.2^47^, using the glmmTMB package^48^. Differences in morphological variables were evaluated by means of multivariate analysis of variance (MANOVA), followed by univariate ANOVAs, with cultivar as fixed effect. We excluded the distance from this analysis because of unbalanced samples and collinearity problems. For this variable we proposed a generalized linear model (GLM) following a Gaussian error distribution. We considered a Bonferroni adjusted alpha level of 0.0125.

Nectar standing crop was assessed by means of GLM following a Gaussian error distribution, with cultivar as fixed effect. To test for differences in nectar production (volume and sugar concentration) we proposed a mixed model (GLMM), following a Gaussian error distribution, with cultivar and flower age as fixed effects, and included branch as random variable to account for data dependency. When we evaluated sugar concentration, only cultivar was considered as fixed effect.

In case there were any differences on the density of pollinators between both selected field zones, we initially included the zone as a fixed effect in the model but removed as it was never significant (p > 0.05). To analyse number of individuals per transect we proposed a GLMM, with a two-way interaction between year and pollinator species, cultivar and the proximity to a bumblebee nest as fixed factors, and transect as random factor, following a negative binomial distribution to account for the overdispersion of the data.

We proposed a GLMM to test the influence of cultivar and pollinator species (fixed factors) on the number of flowers visited per minute, following a negative binomial distribution to account for the overdispersion of the data, and included the log-transformed observation duration as an offset. To evaluate differences in the resource foraged, we proposed a GLMM with resource (nectar/pollen) as response variable, following a Bernoulli binomial error distribution, with year, time of day, and a two-way interaction between species and cultivar (fixed factors), and included the transect as random factor. We analysed a randomly generated sample from the transects dataset, in order to have a balanced sample size of pollinators for each year.

Pollen loads were analysed with a GLMM following a negative binomial error distribution to account for overdispersion, considering a two-way interaction between species and forager type. To evaluate differences among body parts, we repeated the analysis considering a two-way interaction between species and body region, with individual as random factor to account for data dependency, and performed post hoc comparisons across body parts with the emmeans package^49^.

Floral constancy was assessed by means of a GLMM with a Bernoulli binomial error distribution, considering cultivar, pollinator species, time of day (fixed factors), and the number of flowers visited as an offset.

All models were inspected for over-/under dispersion, zero inflation and distribution of the residuals. Scaled residuals were simulated from the fitted model using the DHARMa packages^50^. Significance of the different terms in models was tested starting from the higher-order terms model using *anova* function to compare between nested models^51^. Non-significant terms (p > 0.05) were removed (see Table S2 in Supplementary information).

## Supplementary Information

Supplementary Information.

## Data Availability

The datasets generated for this study are available on request to the corresponding author.
