# Supplementary material for: Managed honeybees and South American bumblebees exhibit complementary foraging patterns in highbush blueberry
Source: Sci Rep. 2021 Apr 14;11:8187. doi: 10.1038/s41598-021-87729-3 (PMC8046787; doi:10.1038/s41598-021-87729-3)
Supplement: Supplementary file 1 — Supplementary Information. [file 41598_2021_87729_MOESM1_ESM.doc]

Supplementary information

Managed honeybees and South American bumblebees exhibit complementary foraging patterns in highbush blueberry

M. Cecilia Estravis-Barcala^a,b^, Florencia Palottini^a,b^, Ivana Macri^a,b,c^, Denise Nery^a,c^, Walter M. Farina^a,b,*^.

^a^Laboratorio de Insectos Sociales, Departamento de Biodiversidad y Biología Experimental, Facultad de Ciencias Exactas y Naturales, Universidad de Buenos Aires, Buenos Aires, Argentina.

^b^Instituto de Fisiología, Biología Molecular y Neurociencias (IFIBYNE), CONICET-Universidad de Buenos Aires, Buenos Aires, Argentina.

^c^Instituto de Ingeniería Rural, Centro de Investigación de Agroindustria (CIA), Instituto Nacional de Tecnología Agropecuaria (INTA), Castelar, Buenos Aires, Argentina.

*Corresponding author: Walter M. Farina

Email: [walter@fbmc.fcen.uba.ar](mailto:walter@fbmc.fcen.uba.ar)

Postal address: Instituto de Fisiología, Biología Molecular y Neurociencias (IFIBYNE), CONICET-Universidad de Buenos Aires, Ciudad Universitaria, C1428EGA, Buenos Aires, Argentina.

**Supplementary Table S1. Floral morphology of blueberry cultivars, ‘Emerald’ and ‘San Joaquin’.** Both varieties presented differences in the three floral traits studied. Means within columns followed by different letters are significantly different at p < 0.0125.

| Cultivar | N | Throat diameter (mm; mean ± SE) | Corolla length (mm; mean ± SE) | Corolla width  (mm; mean ± SE) | Distance between anther and stigma (mm; mean ± SE) |
| --- | --- | --- | --- | --- | --- |
| Emerald | 55 | 3.82 ± 0.06 a | 8.87 ± 0.07 a | 8.50 ± 0.07 a | 1.54 ± 0.05 a |
| San Joaquin | 54 | 3.45 ± 0.05 b | 8.62 ± 0.07 b | 7.67 ± 0.08 b | 1.62 ± 0.06 a |

**a**) **b**)

**Supplementary Fig. S1**. **Nectar sampling of blueberry cultivars, ‘Emerald’ and ‘San Joaquin’**. (**a**) Nectar availability (standing crop) in both varieties. San Joaquin flowers offered a higher volume of nectar. (**b**) Nectar production during the first 3 days after anthesis. San Joaquin flowers presented more nectar than Emerald ones for all ages evaluated. Boxplot shows the median and interquartile range (IQR), with whiskers showing the maximum value within 1.5 IQR, and individual points mark values outside this range. Asterisks indicate statistical differences (**, p < 0.01; ***, p < 0.001). Sample size indicated in brackets.

**Supplementary Table S2**. Set of variables considered in the generalized linear (mixed) models proposed for the characterization of blueberry cultivars ‘Emerald’ and ‘San Joaquin’, and foraging patterns of *Apis mellifera* and *Bombus pauloensis*.

| Section and Model | Coefficient | Std. Error | Z value | P value |
| --- | --- | --- | --- | --- |
| **Characterization of cultivars** | | | | |
| *a) Nectar standing crop*  *a1) Nectar Volume*  Model (Gaussian): Nectar volume ~ cultivar | | | | |
| Fixed effect:  Cultivar (EM/SJ) | 1.09 | 0.37 | 2.94 | **3.3e-03** |
| *a2) Nectar sugar concentration*  Model (Gaussian): Nectar concentration ~ cultivar | | | | |
| Fixed effect:  Cultivar (EM/SJ) | -1.68 | 1.29 | -1.30 | 0.194 |
| *b) Nectar production*  *b1) Nectar volumen*  Model (Gaussian): Nectar volume ~ cultivar + flower age + (1|branch) | | | | |
| Fixed effect:  Cultivar (EM/SJ)  Flower age (2-day-old)  Flower age (3-day-old) | 2.55  4.09  6.88 | 0.51  0.53  0.56 | 4.98  7.68  12.31 | **6.5e-07**  **1.7e-14**  **< 2e-16** |
| *b2) Nectar sugar concentration*  Model (Gaussian): Nectar concentration ~ cultivar + flower age + (1|branch) | | | | |
| Fixed effect:  Cultivar (EM/SJ)  Flower age (2-day-old)  Flower age (3-day-old) | -2.50  0.91  5.93 | 2.12  1.67  1.64 | -1.18  0.54  3.62 | 0.238  0.586  **3.0e-04** |
| **Foraging patterns at population level** | | | | |
| Model^#^ (Negative Binomial):  Nr bees per transect ~ year * species + cultivar + *Bombus* nest + (1 | transect) | | | | |
| Fixed effect:  Year * Species:  2019:Bombus  Cultivar (EM/SJ)  *Bombus* nest (distant/nearby) | 1.26  -0.61  -0.09 | 0.19  0.09  0.08 | 6.81  -6.61  -1.11 | **9.5e-12**  **3.8e-11**  0.268 |
| *# Field zone was initially included as a 2-level fixed effect but was removed from the model (p= 0.6138).* | | | | |
| **Foraging patterns at individual level** | | | | |
| *a) Number of flowers visited*  Model (Negative binomial): Nr flowers ~ cultivar + species + offset(log(duration)) | | | | |
| Fixed effects:  Cultivar (EM/SJ)  Species (Apis/Bombus) | -0.17  0.40 | 0.06  0.07 | -2.76  6.73 | **5.7e-03**  **1.8e-11** |
| *b) Resources foraged*  Model (Bernoulli): Resource ~ year + time of day + cultivar * species + (1|transect) | | | | |
| Fixed effects:  Year (2017/2019)  Time of day (Morning/Afternoon)  Cultivar*Species:  SanJoaquin: Bombus | 0.15  0.17  -1.31 | 0.26  0.26  0.58 | 0.586  0.67  -2.24 | 0.558  0.502  **0.025** |
| *c) Pollen from body*  *c1) by forager type*  Model (Negative binomial): Nr tetrads ~ forager type * species | | | | |
| Fixed effects:  Forager type (Nectar/Pollen)  Species (Apis/Bombus)  Forager type * Species | 1.35  2.92  -0.68 | 0.49  0.50  0.71 | 2.77  5.85  -0.95 | **0.0057**  **4.9e-09**  0.3402 |
| *c2) by body region*  Model (Negative binomial): Nr tetrads ~ species * body region + (1|ID) | | | | |
| Fixed effects:  Species * Body region:  Bombus:Legs  Bombus:ThoraxAbdomen | -1.14  -2.53 | 0.70  0.69 | -1.64  -3.69 | 0.101  **2.3-e04** |
| *d) Floral constancy*  Model (Bernoulli): Constancy ~ species + cultivar + offset(log(nr.flowers)) | | | | |
| Fixed effects:  Species (Apis/Bombus)  Cultivar (EM/SJ)  Time of day (Morning/Afternoon) | -0.97  -0.12  -0.01 | 0.47  0.50  0.48 | -2.06  -0.25  -0.02 | **0.0396**  0.8031  0.9836 |

**Supplementary methods**

*Characterization of blueberry cultivars*

To evaluate the floral morphology of both cultivars, we collected one recently opened flower from one inflorescence per plant for morphometric measurements during the 2019 season (N = 55 Emerald, N= 54 San Joaquin). The collected flowers were preserved in 95% ethanol for later measurements in the laboratory. Following the methodology described by Courcelles and collaborators [1], we measured the following variables using a digital calliper: the total length of the flower from the base of the floral tube to the opening of the corolla, the corolla throat (opening) diameter, and the width of the corolla at the widest point. With the aid of a scalpel, we cut a slit in the corolla to measure the distance between the anthers and the stigma while those organs were still *in situ*.

Additionally, to characterize the nectar availability (i.e. standing crop) of ‘Emerald’ and ‘San Joaquin’ flowers in 2017, we extracted nectar of freshly clipped flowers (a single flower per plant; N = 59 ‘Emerald’, N = 47 ‘San Joaquin’) with 5-μl graduated capillary glass tubes, taking precautions to avoid damaging any floral structures. We computed the volume of nectar as the length of the nectar column in the capillary tube, and the sugar concentration on a % weight on weight (henceforth: w/w) basis (sucrose equivalents; g sucrose per 100 g solution) was estimated with the help of a pocket refractometer (Carl Zeiss, accuracy: 0–85 ± 0.5%).

Nectar production for both cultivars was assessed during the 2019 season. We protected inflorescences from visitors covering them with voile bags before anthesis [2] in 11 ‘Emerald’ and 10 ‘San Joaquin’ plants (one branch per plant). During four consecutive days, we marked all newly opened flowers to monitor the age throughout their life (corolla drops after four days) in order to be able to measure nectar volume and sugar concentration of open flowers of all ages on two consecutive days at the same time on different plants. Each flower was sampled only once and the flower age was recorded, using untouched flowers for each new measurement.

*Processing of pollen samples from bees*

To quantify the pollen on each body part, the dissected body region was placed in separate microcentrifuge tubes containing 0.5 ml of hexane (or 0.5 ml of 96% ethanol in the case of the thorax-abdomen). Each tube was vortexed for 30 s, sonicated in a water bath for 30 min (Ultrasonic cleaner Testlab tb02, 40 Khz), and then vortexed a second time. The bee parts were removed from each tube and the pollen solution was centrifuged for 1 min at 5,000 rpm. The supernatant was removed, replaced with ethanol, and stored until pollen counts were conducted. Just prior to counting, the samples were again centrifuged for 30 s at 10,000 rpm and the supernatant removed, leaving 0.03–0.04 ml of pollen solution in each tube. Each tube was stirred briefly with a pipette tip before two aliquots of 10 μl of solution each were loaded into the counting chambers of a metallized hemacytometer (Marienfeld Neubauer-improved) and the pollen grains counted. We recorded the number of tetrads within the whole counting grid, since blueberry pollen is considerably larger than blood cells (35–71 μm vs 6–8 μm). We then calculated the total pollen load per sample considering the volume of both counting chambers (informed by the hemacytometer manufacturer) and the volume of the total pollen solution sampled. For blueberry, the ‘unit’ was the 4-grain pollen tetrads and pollen identity was confirmed with reference samples of both cultivars. The abundance of other pollen types was disregarded.

References

1. Courcelles, D. M. M., Button, L., & Elle, E. Bee visit rates vary with floral morphology among highbush blueberry cultivars (*Vaccinium corymbosum* L.). *J. Appl. Entomol.* **137**(9), 693-701 (2013).
2. Corbet, S. A. Nectar sugar content: estimating standing crop and secretion rate in the field. *Apidologie* **34**(1), 1-10 (2003).
